# Nuclear Translocation of B-Cell-Specific Transcription Factor, BACH2, Modulates ROS Mediated Cytotoxic Responses in Mantle Cell Lymphoma

**DOI:** 10.1371/journal.pone.0069126

**Published:** 2013-08-02

**Authors:** Zheng Chen, Eric F. Pittman, Jorge Romaguera, Luis Fayad, Michael Wang, Sattva S. Neelapu, Peter Mclaughlin, Larry Kwak, Nami McCarty

**Affiliations:** 1 Center for Stem Cell and Regenerative Disease, Brown Foundation Institute of Molecular Medicine for the Prevention of Human Diseases (IMM) University of Texas-Health Science Center at Houston, Houston, Texas, United States of America; 2 Department of Lymphoma and Myeloma, the University of Texas MD Anderson Cancer Center, Houston, Texas, United States of America; University of Navarra, Center for Applied Medical Research, Spain

## Abstract

BACH2, a B-cell specific transcription factor, plays a critical role in oxidative stress-mediated apoptosis. Bortezomib (Velcade^TM^) is widely used to treat relapsed mantle cell lymphoma (MCL) patients despite varying clinical outcomes. As one of the potential mechanisms of action, bortezomib was reported to elicit endoplasmic reticulum (ER) stress which triggers reactive oxygen species (ROS). In the present study, we investigated the redox-sensitive intracellular mechanism that might play a critical role in bortezomib response in MCL cells. We demonstrated that in MCL cells that are sensitive to bortezomib treatments, BACH2 was translocated to the nucleus in response to bortezomib and induced apoptotic responses through the modulation of anti-oxidative and anti-apoptotic genes. On the other hand, in bortezomib resistant cells, BACH2 expression was confined in the cytoplasm and no suppression of antiapoptotic or antioxidative genes, Nrf2, Gss, CAT, HO-1 and MCL1, was detected. Importantly, levels of BACH2 were significantly higher in bortezomib sensitive MCL patient cells, indicating that BACH2 levels could be an indicator for clinical bortezomib responses. BACH2 translocation to the cytoplasm after phosphorylation was inhibited by PI3K inhibitors and combinatory regimens of bortezomib and PI3K inhibitors sensitized MCL cells to bortezomib. These data suggest that cellular distribution of BACH2 in response to ROS determines the threshold for the induction of apoptosis. Therapies that inhibit BACH2 phosphorylation could be the key for increasing bortezomib cytotoxic response in patients.

## Introduction

Mantle Cell Lymphomas (MCL), a rare but particularly deadly sub-type of Non-Hodgkin's Lymphoma (NHL), are refractory to conventional therapies and display cellular heterogeneity and genomic instability [1]–[3]. The major genetic alteration in MCL that distinguishes them from low-grade B cell lymphomas is the t(11;14)(q13;q32) translocation, leading to increased levels of cyclin D1 (CCND1) gene expression [1], [2]. Although this translocation is a genetic hallmark of most MCL, CCND1 overexpression is not sufficient to induce MCL [4], [5], suggesting that other genetic events, possibly acting cooperatively with CCND1 overexpression, are required for the initiation and progression of MCL. Clinical features of MCL such as sites of involvement in the body (e.g. bone marrow, lymph nodes, blood and gastrointestinal system), being refractory to standard chemotherapies, frequent patient relapses, short median survival (∼3 years) and number of deaths suggest that MCL is a difficult-to-treat type of NHL which needs a significant advancement in understanding its major oncological signaling pathways with the prospect of identifying novel potential therapeutic targets [6].

Bortezomib (Velcade®), which is a reversible inhibitor of the 26 S proteasome, first gained FDA approval as a single-agent treatment in patients with relapsed or refractory MCL [7]–[10]. Bortezomib inhibits the ubiquitin-proteasome pathway and alters multiple cellular signaling cascades, including those regulating cell growth, differentiation and survival [11], [12]. For example, proteasome inhibition prevents the degradation of pro-apoptotic factors, which facilitates the activation of programmed cell death in neoplastic cells [13]; however, the precise mechanisms of action are controversial.

Because of varying clinical outcomes against bortezomib, several efforts including our own, have been made to identify the mechanism of bortezomib resistance in hematological malignancies, including MCL and other tumors [14]–[16]. As one of the potential mechanisms of action, bortezomib was reported to elicit the unfolded protein response (UPR), which is activated when the physiological environment of the ER is altered [17]–[19]. The induction of ER stress induces reactive oxygen species (ROS), which affects treatment responses to bortezomib in MCL [19] and multiple myeloma (MM) [20]. Therefore, in the present study, we aim to determine the redox-sensitive intracellular mechanism that might play a critical role in bortezomib response in MCL cells.

BACH2, a B-cell specific transcription factor, and a member of the CNC family of proteins, binds to the Maf recognition element (MARE) and/or antioxidant response element (ARE) by forming homodimers or dimerizing with small Maf transcription factors [21], [22]. BACH2 has been shown to play a critical role in oxidative stress-mediated apoptosis induced by cytotoxic agents in lymphoma cells [23]. Recently, reports have also shown that BACH2 expression level is correlated with overall disease-free survival in diffuse large B-cell lymphoma (DLBCL) patients [24], indicating a tumor suppressive role of BACH2. In this study, we demonstrate that MCL cells that are resistant to bortezomib (Mino and Rec-1), showed lower levels of BACH2 than the bortezomib-sensitive MCL cells (Jeko and SP53). This differential response of MCL cells was not found to be attributed to the level of reactive oxygen species induced by bortezomib treatment. Instead, subcellular localization of BACH2 determined apoptotic response to bortezomib. In bortezomib sensitive Jeko and SP53 MCL cells BACH2 was translocated into the nucleus, which was not observed in bortezomib-resistant cells, Mino and Rec-1. BACH2 nuclear translocation determined apoptosis induction through differential modulation of antioxidative and prosurvival/antiapoptotic genes.

In summary, we discovered that BACH2 activity is a critical determinant for cellular response to bortezomib in MCL. Understanding how these processes are molecularly coordinated will be the key to resolving drug responses in MCL.

## Materials and Methods

### Reagents and antibodies

2,7-dichlorodihydrofluorescein-diacetate (DCH-FDA, #35845-1G), N-acetyl-L-cysteine (NAC, #A7250), and leptomycin-B, #L2913) were purchased from Sigma Chemical (St Louis, MO). Cell Titer blue viability kit (#G8080) was from Promega (Madison, WI), Draq5, #4084). cDNA synthesis kit (#18080200) was from Invitrogen and mini-RNA isolation kit (#74104) from Qiagen. Primary anti-BACH2 antibody (#ab83364), anti-β-actin (#ab15263), anti-TATA binding protein (anti-TBP, #ab63766–100) and HRP-conjugated secondary antibodies (#ab6721) were purchased from Abcam (Cambridge, MA). PI3K inhibitor (LY-294002) (#L9908), bruton's tyrosine kinase inhibitor (PCI-32765, #S2680) and CAL-101 (#S2226) were purchased from Selleckchem.

### Patient samples

The tissue samples were provided by the University of Texas M. D. Anderson Cancer Center Satellite Lymphoma Tissue Bank which was supported by Institutional Core Grant # NCI/NIH - CA16672 and MDACC Lymphoma SPORE Grant # NCI/NIH – P50CA136411. Blood specimens from MCL patients were obtained after informed consents, as approved by M.D. Anderson Cancer Center as well as by the University of Texas-Health Science Center Institutional Review Boards. All participants were provided a written informed consent to participate in the study. All primary patient peripheral blood mononuclear cells (PBMC) were isolated from aphaeresis blood by standard Ficoll gradient methods. All patient samples were diagnosed with MCL at the time of collection based on t(11;14) translocation, cyclin D1 reactivity and were in the leukemic phase at the time of aphaeresis.

### Cell culture

MCL cell lines (Jeko, SP53, Mino and Rec-1), a Burkitt lymphoma cell line (Raji) and human embryonic kidney 293T cells were obtained from American Type Culture Collection (Manassas, VA). MCL cells and Raji were cultured in the RPMI 1640 medium with 10% heat-inactivated fetal bovine serum (FBS), 100 µg/mL streptomycin, and 100 µg/mL penicillin. 293T cells were cultured in DMEM with MEM non-essential amino acids, 10% heat-inactivated fetal bovine serum (FBS), 100 µg/mL streptomycin, 100 µg/mL penicillin, 4.00 mM L glutamine, 1000 mg/L glucose, and 110 mg/L sodium pyruvate.

### Cell viability assay

MCL cell viability was determined by their metabolic ability to reduce resazurin into resorufin dye (CellTiter-Blue, Promega) as described previously in our reports [25], [26]. Briefly, cells (2×10^4^) were suspended in complete medium (100 µl) per well followed by treatment with bortezomib (diluted in 100 µl complete medium) to achieve desired concentration range (1–100 nM) for 24 h at 37°C. Control or untreated wells were washed with fresh complete medium followed by resazurin dye incubation for 3 h at 37°C according to manufacturer's instruction. The fluorescent signal was measured at Ex/Em (560/590 nm) using a fluorescence plate reader equipped with SoftMax Pro software (Molecular Devices). Percentage viability was calculated by Ft (Fluorescence intensity of bortezomib-treated cells)/Fc (Fluorescence intensity of control cells) ×100. This experiment was performed in quadruplicate for each treatment. Percentage cytotoxicity was calculated by comparing with control as 100%. Results are expressed as mean ± standard deviation (SD). To study the synergistic effect of kinase inhibitors with bortezomib, cells were treated with PI3K inhibitors (20 µM, LY294002) or CAL-101 (5 µM) with bortezomib.

### Assessment of reactive oxygen species (ROS)

Bortezomib-induced intracellular generation of ROS in MCL cells was assessed by employing a specific cell permeable fluorescent probe, DCH-FDA, as described previously [27]. Briefly, cells (2×10^5^ cells) were incubated in complete medium with or without bortezomib (20 nM) for 16 h, followed by labeling of cells with DCH-FDA (10 µM) in PBS for 20 min at 37°C in the dark. The mean fluorescence intensity (MFI) of samples was then measured at Ex/Em, 480/520 nm and then analyzed by using fluorescence-activated cell sorting LSRII flow cytometer (BD Biosciences, NJ). Results were expressed by calculating relative fluorescence intensity (RFI) using the equation: RFI  =  MFI of treated samples (Ft)/MFI of control (Fc) ×100.

### Confocal microscopy and image analysis

The cytoplasmic and nuclear localization of BACH2 were studied by immunostaining followed by confocal microscopy (TCS SP5 laser scanning confocal microscope, Leica). Briefly, control and bortezomib-treated MCL cells were washed with ice-cold sterile PBS (10 mM, pH∼7.4). An aliquot (100 µl, 2×10^4^) of cell suspension was layered onto glass slides at 800 rpm for 15 min (Cytospin 4, Thermo Scientific). Cells were incubated with primary anti-BACH2 antibody (diluted at 1∶500 in blocking buffer) in a humidified chamber at room temperature for 1h followed by three washings with PBS, each for 5 min. Alexa 594-conjugated secondary (1∶200 in blocking buffer for 1 h) was used to visualize BACH2 protein. Draq5^TM^ (Abcam) was used to counter stain the nucleus. Each slide was examined for the presence of BACH2 and Draq5 (nucleus) at 594 nm and 633 nm excitations and the data were compared pixel by pixel. Image acquisition of each slide was done at the same parameters of confocal microscopy using sequential mode for BACH2 and Draq5. To quantitate the BACH2 fluorescence in cytoplasm and nucleus, image analysis software ‘AMIRA’ was used [28]. Briefly, three to five images for each sample were analyzed. An individual image for each channel (red or blue fluorescence) was fed into the software to de-segment the cytoplasm and nuclear material using ‘Ligand Field’. A constant threshold in control and treated samples was used for the cytoplasmic and nuclear demarcation for these analyses. Using material statistics built into the software, fluorescence intensity was measured. Results were expressed as the mean of three samples for cytoplasmic and nuclear localization. Non-specific fluorescence, if any, was excluded using a de-selection mode of the software.

### Quantitative reverse transcription-polymerase chain reaction (qRT-PCR)

Total RNA from MCL cell lines, normal B-cell and primary MCL cells was extracted using RNAeasy Mini kit (Qiagen). RNA was eluted in DEPC-treated water supplied with kit. Quantification was done by taking absorbance at 260/280 nm ratio using Nanodrop (Eppendorf BioPhotometer plus, Hamburg, Germany). Equal amount of RNA was used for cDNA synthesis using SuperScript II First-Strand Synthesis System (Invitrogen, CA). The following primers were used for qRT-PCR; ***Nrf2*** sense 5′ GCAATGAAGACTGGG CTCTC 3′, antisense 5′ AAACCAGTGGATCTGCCAAC 3′, ***Gss*** sense 5′ TACGGCTCACCCAATGCTC 3′, ***Gss*** antisense 5′ CTATGGCACGCTGGTCAAATA 3′, ***CAT*** sense 5′ TGGAGCTGGTAACCCAGTAGG 3′, ***CAT*** antisense 5′ CCTTTGCCTTGGAGTATTTGGTA 3′, ***HO-1*** sense 5′ AAGACTGCGTTCCTGCTCAAC 3′, ***HO-1*** antisense 5′AAAGCCCTACAGCAACTGTCG 3′, ***MCL1*** sense 5′ GTGCCTTTGTGGCTAAACACT 3′, ***MCL1*** antisense 5′ AGTCCCGTTTTGTCCTTACGA 3′. qRT-PCR was performed using SYBR Green/ROX qPCR master mix (Qiagen, CA). Human β-actin gene, sense 5′ GGACTTCGAGCAAGAGATGG 3′, antisense 5′AGCACTGTGTTGGCGTACAG3′, was used as an internal control.

### Immunoblottings

Whole cell lysates, cytoplasmic and nuclear extracts were prepared by using ACTIVE MOTIF^TM^ kit (Carlsbad, CA) according to the Manufacturer's instruction. Cytosolic and nuclear fractions of MCL cells were separated using the kit employing a hypotonic buffer method. Protein was estimated by Bradford dye reagent (BIO-RAD, Hercules, CA) by taking absorbance at 550 nm using spectrophotometer (Molecular Devices). An equal amount of protein (100 µg) was loaded and separated on SDS-PAGE (6% or 10%). Proteins were transferred onto nitrocellulose membrane overnight at 25 V at 4°C. Membranes were probed with polyclonal anti-BACH2 antibodies (Abcam, MA) followed by HRP-labeled secondary antibodies. Proteins were visualized by chemiluminescence imager (Fluor Chem-M, Cell Biosciences, CA). β-Actin and TATA-binding protein (TBP) were used as cytoplasmic and nuclear loading controls respectively. Raji cells were used as a negative control for BACH2 protein. Band densitometry was performed using NIH J-Image software.

### Assessment of apoptosis

For the assessment of cell viability and both early and late-stage apoptosis, cells were stained with the Annexin-V kit and the 7-AAD Kit (e-Biosciences, CA). Briefly, cells were washed twice in cold PBS, resuspended in binding buffer (1×10^6^ cells in 0.1 ml), and 10 µl of FITC-conjugated Annexin-V and 20 µL of 7-AAD were added. Cells were incubated for 15 min in the dark, an additional 400 µL of binding buffer were added, and the cells were analyzed within 1 hour by flow cytometry. Acquisition was performed on a FACS LSRII (BD Biosciences, CA).

### Statistical analysis

The results were expressed as the mean ± SD of the data collected from three or four independent experiments or mean ± SEM (standard error of the mean). Statistical significance was determined by Student's t-test (p-value <0.05 considered significant).

### Drug combination assay

The synergic cytotoxic effects of bortezomib and conventional combination chemotherapeutic regimens were determined by combination index (CI) method based on Chou and Talalay equation [29], and analyzed by the CompuSyn software (ComboSyn, NJ, USA). Briefly, Combination index (CI) equation is quantitative measure of the degree of drug interaction in term of synergism and antagonism of a given endpoint of the effect measurement [30], and the following median-effect equation [*f_a_/f_u_  =  (D/D_m_)^m^* ; a general equation for dose-effect relationship that takes into account both the potency (*D_m_*) and the shape (*_m_*) of dose-effect curve where *f_a_* and *f_u_* are the fractions affected and unaffected, respectively [31] is the basis of following CI equation:
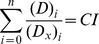



In this equation, *n* is the number of combined drugs; (*D_x_)i* is the dose of Drug *i* alone that inhibits *x*%; and (*D*)*i* is the portion of Drug *i* in drug combination also inhibits *x*%. Synergy is present when the CI is less than 1.0, additive effect is when CI equals 1.0, and antagonism is when CI greater than 1.0.

## Results

### Bortezomib resistant MCL patient cells express lower levels of BACH2 compared to bortezomib sensitive MCL patient cells

**Figure 1:**
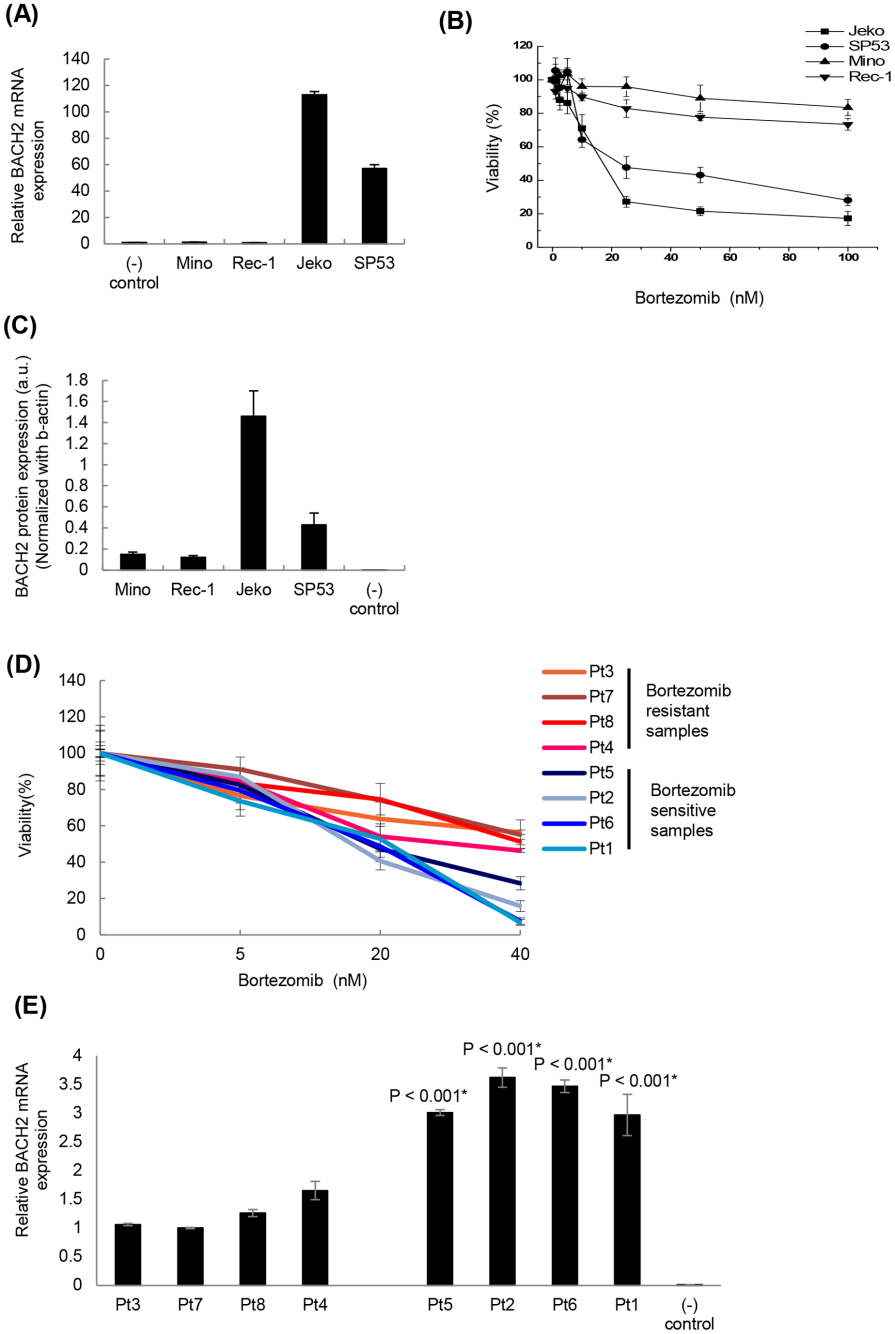
Lower levels of BACH2 -mRNA and -protein are expressed in bortezomib resistant MCL cell lines and patient cells compared to bortezomib sensitive MCL cell lines and patient cells. (**A**) mRNA levels of BACH2 in MCL cell lines were determined by qRT-PCR using SYBR green with the 7900 ABI system. Fold of BACH2 levels in Jeko, SP53, Mino, and Rec-1 was calculated by ΔΔCt method considering Ct value in Raji as a negative control. Data represents mean ± SD of three independent experiments. (**B**) Dose dependent bortezomib-induced cytotoxicity at 24 h was determined by resazurin dye reduction assay as described in material and methods. Jeko and SP53 cells were found to be more responsive than Mino and Rec-1 to bortezomib treatment. Data points represent mean of quadruplicates from seven independent experiments. (**C**) Protein levels of BACH2 in whole cell lysates of indicated MCL were determined by immunoblotting. Actin was used as a protein loading control. Raji cell lysate was used as a negative control. BACH2 band density was normalized with β-actin using NIH J-image software. After normalization with β-actin, Jeko and SP53 have higher BACH2 levels compared to Mino and Rec-1. Data represent three independent experiments. (**D**) Dose dependent bortezomib-induced cytotoxicity at 24 h was determined by MTT assay using primary MCL patient cells. All primary patient peripheral blood mononuclear cells (PBMC) were isolated from aphaeresis blood by standard Ficoll gradient methods. Data represent three independent experiments. (**E**) BACH2 mRNA levels were measured by real-time PCR using different MCL patient samples in Figure 1D. Data represent three independent experiments.

Since higher BACH2 expression levels are associated with a better prognosis in large B cell lymphomas, we first examined levels of BACH2 using different MCL cell lines (Jeko, SP53, Mino and Rec-1) using quantitative real time PCR. Compared to Mino and Rec-1 MCL cell lines, Jeko and SP53 MCL cell lines showed significantly increased BACH2 mRNA (∼50–100 folds) levels by real time PCR (**Figure 1A**). To find the correlation between BACH2 levels and bortezomib response, we then measured the cytotoxic response of Jeko, SP53, Mino and Rec-1 to bortezomib (0–100 nM/24 h) using MTT assays. Results showed dose dependent decrease in the viability of MCL cells. Jeko and SP53 cells were significantly more sensitive to bortezomib compared to Mino and Rec-1. IC_50_ for Jeko and SP53 was estimated to be less than ∼15 nM at 24 h. Typically, Mino and Rec-1 at 20 nM bortezomib treatment maintained ∼80–90% viability. Moreover, up to 100 nM bortezomib (highest concentration studied), Mino and Rec-1 did not show a significant decrease in viability compared to Jeko and SP53 (**Figure 1B**).

BACH2 protein levels in these cell lines were further studied by immunoblotting. Since Raji cells do not express BACH2 protein it was used as a negative control [23]. Immunoblotting results revealed increased levels of BACH2 protein in Jeko and SP53 compared to Mino and Rec-1 MCL cells (**Figure 1C** **, Figure S1A**). We then investigated BACH2 levels using MCL patient cells. Cell cytotoxicity after Bortezomib treatment was first measured using MTT assays (**Figure 1D**) and BACH2 mRNA levels were evaluated in each patient cell. The average of IC50 values of bortezomib resistant cells was 53.0 nM and the average of IC50 values of bortezomib sensitive cells was 14.5 nM (**Figure S1B**). Average BACH2 mRNA expression levels were statistically higher in bortezomib sensitive patient cells compared to bortezomib resistant patient cells (**Figure 1E** **and Figure S1C**). Collectively, these results clearly showed that BACH2 levels are higher in bortezomib sensitive MCL cells compared to those in bortezomib resistant MCL cells.

### Bortezomib-induced MCL cytotoxicity involves oxidative stress mediated-apoptosis

**Figure 2:**
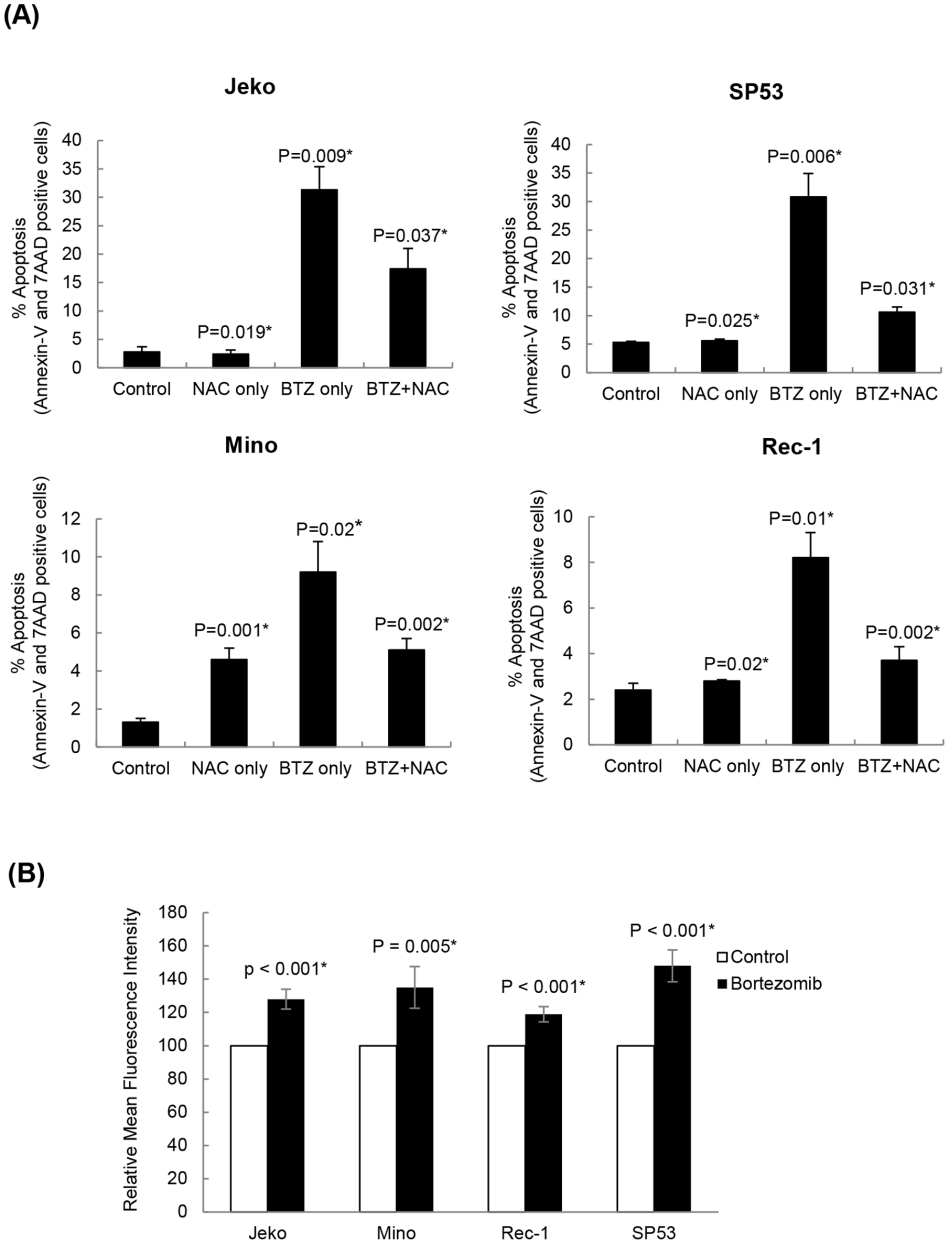
Bortezomib-induced anti-MCL activity involves oxidative stress-mediated apoptosis. (**A**) Bortezomib-induced apoptosis and its inhibition by pretreatment of cells with a ROS scavenger, N-acetyl-L-cysteine (NAC). Apoptosis was determined by Annexin-V/7-AAD dual staining method using FACS. Cells that showed positive staining for both Annexin-V/7AAD were considered apoptotic/late apoptotic. Error bars indicate the standard error of the means (SEM) from two independent experiments. Statistical significance is indicated as (*) for *p*>0.05 and (**) for *p*>0.01 as compared to the respective untreated control. (**B**) Bortezomib-induced ROS generation in MCL cells. Cells were treated for 24 h with 20 nM of bortezomib for fluorimetric assessment of a ROS-specific cell permeable probe, DCH-FDA by FACS as described in materials and methods. After DCH-FDA labeling (10 µM for 20 min/37°C), cells were washed and resuspended in PBS. Mean fluorescence intensities of control and bortezomib-treated samples were determined by FACS. Results are expressed as relative fold of increase in MFI compared to control.

To study the mechanism of cell death involved in bortezomib-induced cytotoxicity in MCL cells, we assessed apoptotic induction by Annexin-v/7AAD dual staining assay using FACS analysis. Results showed that bortezomib treatment (20 nM for 24 h) induced a higher fold of apoptosis in Jeko and SP53 (∼5–7 folds) than in Mino and Rec-1 (∼2–3 folds) as compared to their respective controls (**Figure 2A**). Since previous reports have shown that oxidative stress acts as a key mediator of bortezomib-induced apoptosis in a variety of tumor models [27], [32], we analyzed percentage apoptosis in MCL cells after pretreating cells with a ROS scavenger, N-acetyl-L-cysteine. Compared to bortezomib treated samples, 1h pretreatment of NAC (100 µM) significantly reduced bortezomib-induced apoptosis in MCL cells (**Figure 2A**). Taken together, these results suggest critical role of ROS in apoptotic activity of bortezomib in MCL.

### Bortezomib response in MCL cell lines do not correlate with induced ROS level

The above results suggested the involvement of oxidative stress in bortezomib-induced apoptosis (**Figure 2A**). Therefore, we tested whether observed differential cytotoxic response of Jeko and SP53 compared to Mino and Rec-1 is correlated with the magnitude of ROS induced by bortezomib. We determined the intracellular level of ROS in MCL cells after bortezomib treatment by an ROS-specific fluorescent probe (DCH-FDA) using FACS. Results showed that both bortezomib-sensitive (Jeko and SP53) and bortezomib-resistant (Mino and Rec-1) MCL cells showed generation of ROS after 16hrs of bortezomib treatment (**Figure 2B**). Even though the amounts of ROS generated after bortezomib treatment were statistically significant compared to untreated samples, fold of ROS among cell lines did not significantly differ to each other. These results suggest that equivalent amounts of ROS were generated in these four cell lines in response to bortezomib, therefore other ROS-sensitive intracellular mechanisms might exist to explain differential responses to bortezomib.

### Bortezomib treatment induces nuclear translocation of BACH2

**Figure 3:**
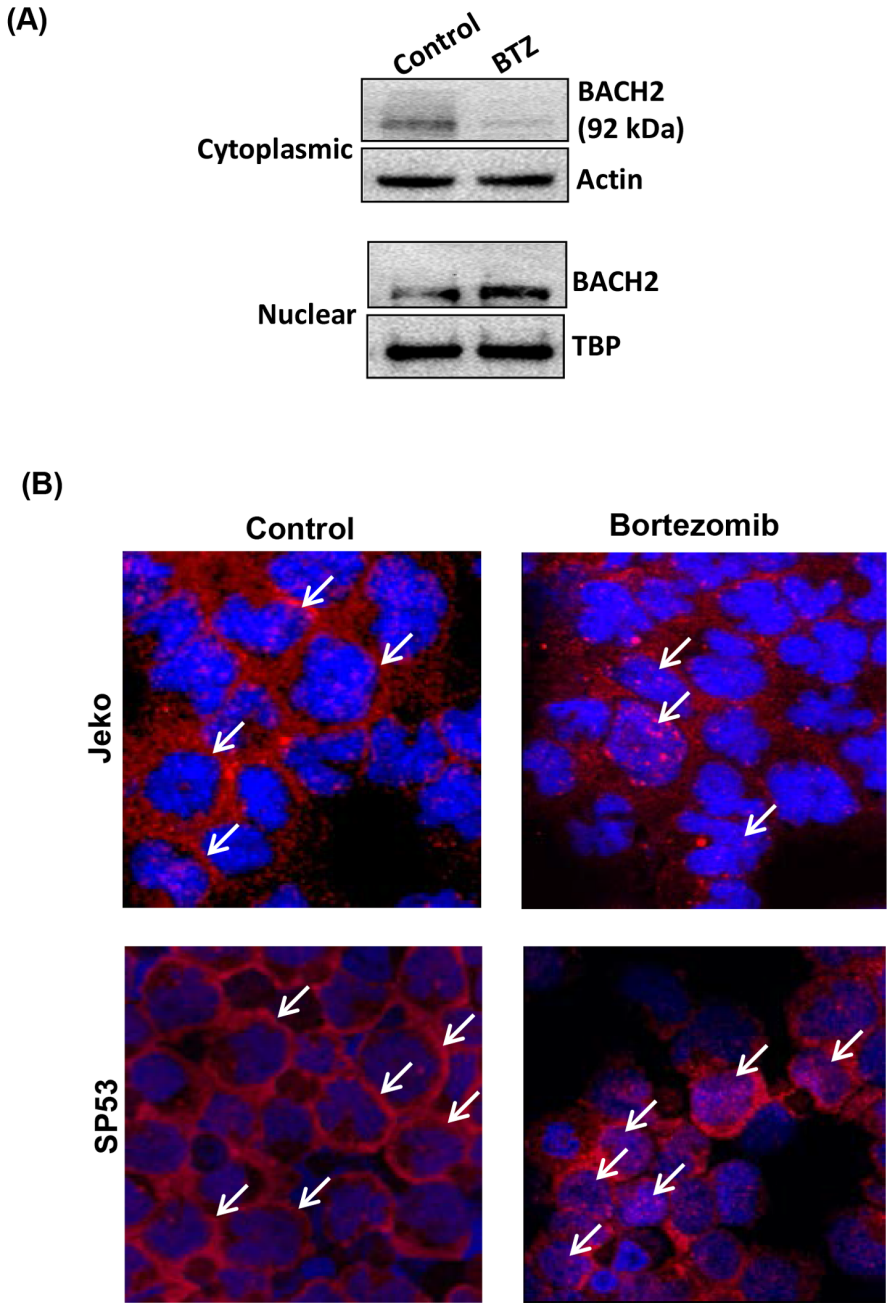
Bortezomib treatment alters subcellular localization of BACH2 in MCL cells. (**A**) Immunoblotting of cytoplasmic and nuclear fraction of control and bortezomib treated Jeko cells. Upper and lower panels show that BACH2 was present in both the cytoplasm and nucleus of untreated cells. Bortezomib treatment decreased the BACH2 level in the cytoplasm and increased it in nuclear fractions compared to untreated control. Actin and TBP were used to normalize the cytosolic and nuclear proteins loading per lane. Data represent four independent experiments. (**B**) Representative confocal microscopic images of control and bortezomib-treated Jeko and SP53 cells. To visualize BACH2 protein in cytoplasm and nucleus, immunocytochemistry samples were prepared as described in the material and methods section. BACH2 fluorescence (red) in control samples was mainly limited to the cytoplasm while bortezomib treatment (20 nM/24h) induced nuclear translocation of BACH2 protein in the nucleus (blue) of Jeko cells. Data represent three independent experiments.

**Figure 4:**
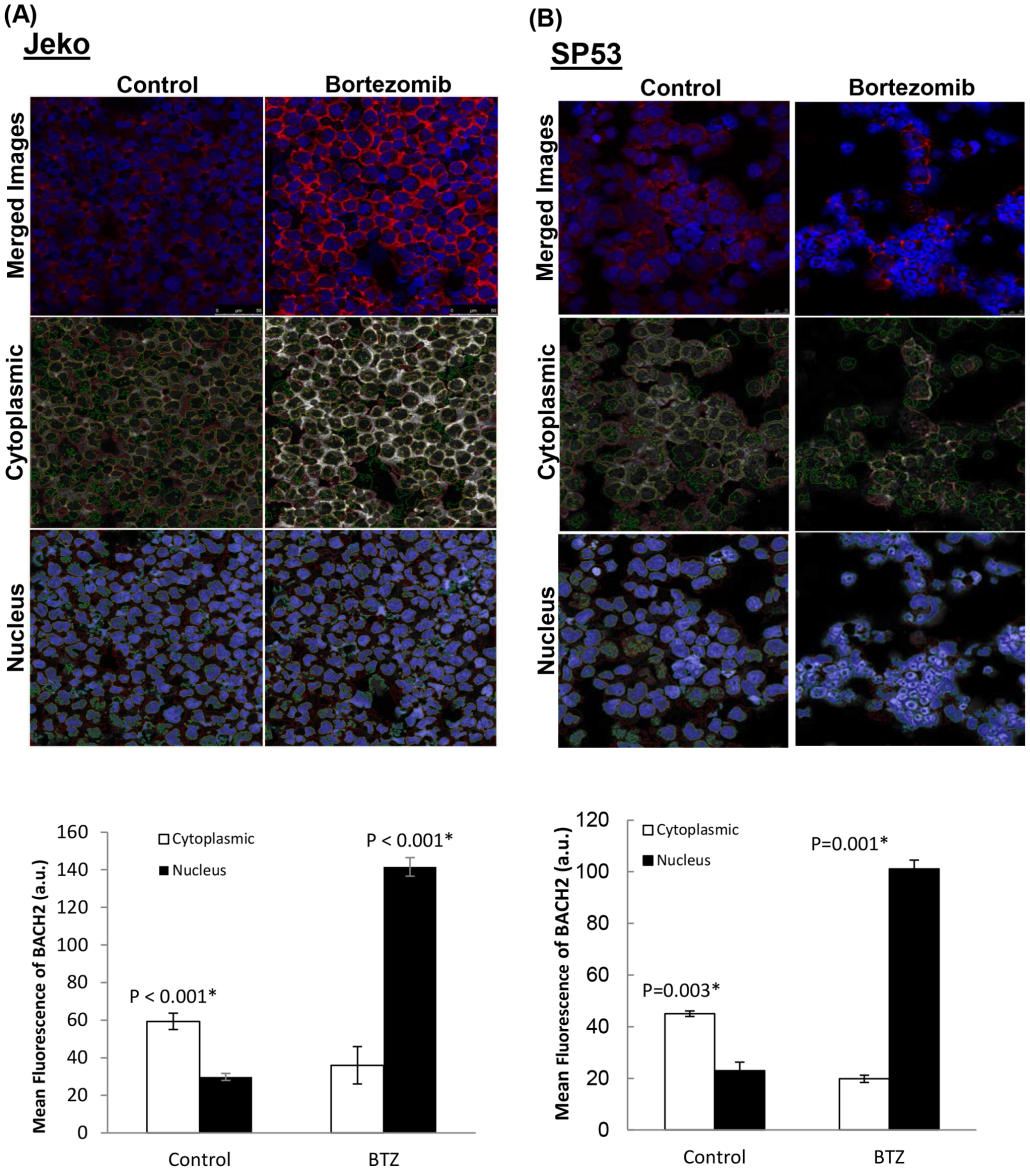
Bortezomib treatment increases nuclear localization of BACH2 in bortezomib susceptible cells. Using ‘AMIRA’ software, confocal images of Jeko (A) and SP53 (B) cells were analyzed to quantitate the BACH2 fluorescence in the cytoplasm and nucleus of control and treated cells. Three random images were analyzed for each cell line. Cytoplasmic and nuclear gating of fluorescence was done using de-segmentation analysis of software using confocal images of both the channels (BACH2, red and Draq5, blue). Representative gated images for cytoplasmic and nuclear marking of control and bortezomib samples for each cell line were shown. Data were collected from analysis of three images and expressed as mean ± SD.

**Figure 5:**
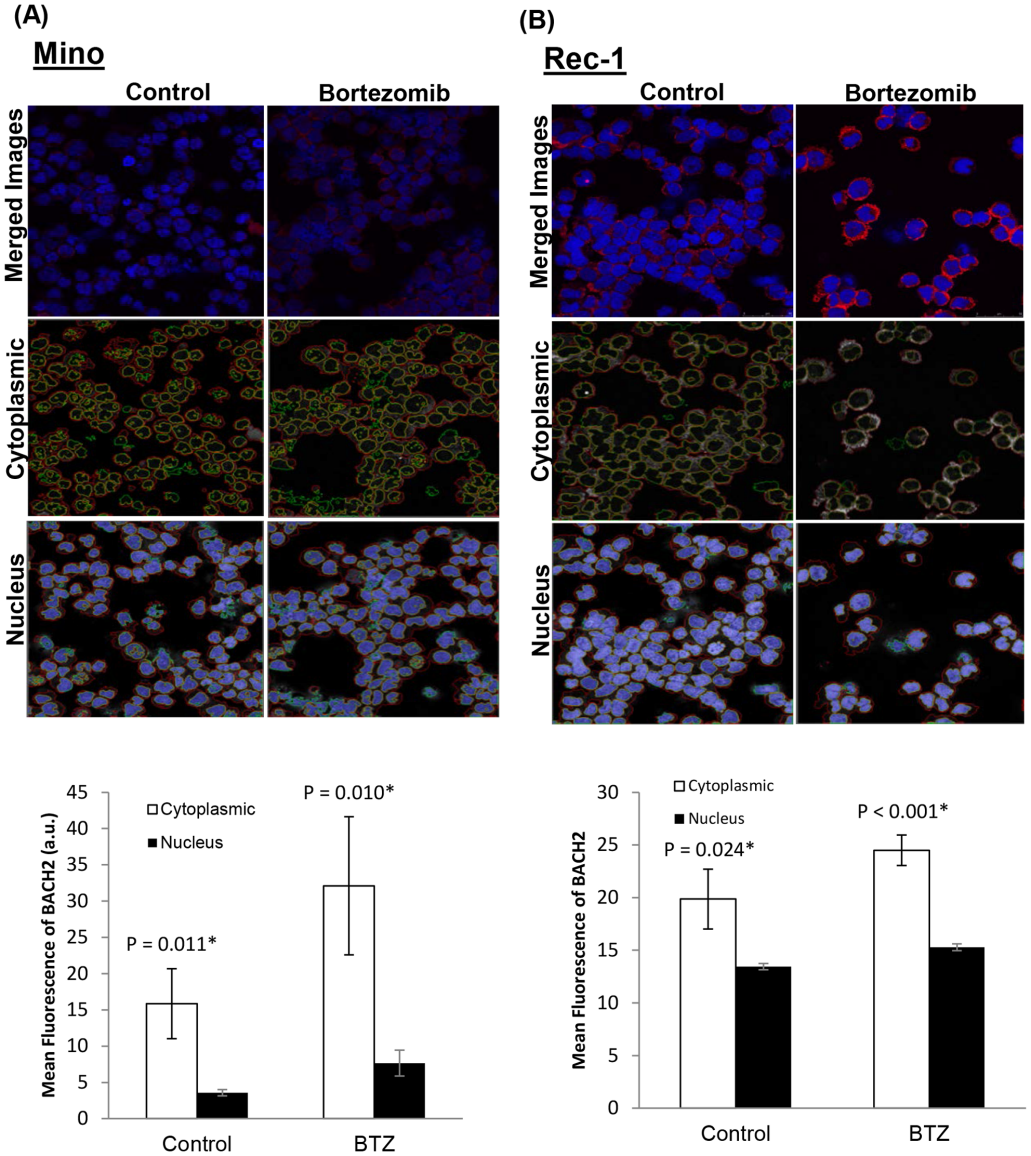
Bortezomib treatment did not increase nuclear localization of BACH2 in bortezomib resistant cells. Using ‘AMIRA’ software, confocal images of Mino (A) and Rec-1 (B) cells were analyzed to quantitate the BACH2 fluorescence in the cytoplasm and nucleus of control and treated cells. Three random images were analyzed for each cell line. Cytoplasmic and nuclear gating of fluorescence was done using de-segmentation analysis of software using confocal images of both the channels (BACH2, red and Draq5, blue). Representative gated images for cytoplasmic and nuclear marking of control and bortezomib samples for each cell line were shown. Data were collected from analysis of three images and expressed as mean ± SD.

Since BACH2 plays important roles in ROS-mediated apoptosis responses, we tested whether BACH2 is translocated into the nucleus upon bortezomib treatment in response to oxidative stress. Therefore, experiments were performed to determine the possibility of BACH2 redistribution in MCL cells after bortezomib treatment. These results were further validated by immunoblottings of BACH2 protein in cytoplasmic and nuclear fractions of control and bortezomib-treated Jeko cells. Results showed that bortezomib treatment (BTZ) resulted in the higher localization of BACH2 protein in the nuclear fraction of Jeko cells compared to controls (**Figure 3A**). These results were further validated by Confocal microscopy. The results showed that BACH2 fluorescence in untreated controls was mainly limited to the cytoplasm (**Figure 3B**)**,** while bortezomib-treated Jeko cells exhibited foci like structure for BACH2 immunostaining in the nucleus suggesting nuclear translocation of BACH2 (arrow) (**Figure 3B**). We have also employed confocal microscopy in combination with image analysis by ‘AMIRA’ software to quantitate the extent of BACH2 localization in the nucleus of MCL cells after bortezomib treatment. Results showed that bortezomib-susceptible Jeko and SP53 cells exhibited increase in BACH2 translocation after bortezomib treatment (**Figures 4A–B**). However, bortezomib-resistant cells, Mino and Rec-1, did not exhibit a significant increase in BACH2 translocation after bortezomib treatment (20 nM/24 h) (**Figures 5A–B**). Quantification of fluorescence indicates that BACH2 signaling increases in the nucleus after bortezomib treatment in Jeko and SP53, but not in Mino and REC-1 cells (**Figures 4** **and** **5**).

### BACH2 nuclear translocation represses anti-oxidative and anti-apoptotic genes

**Figure 6:**
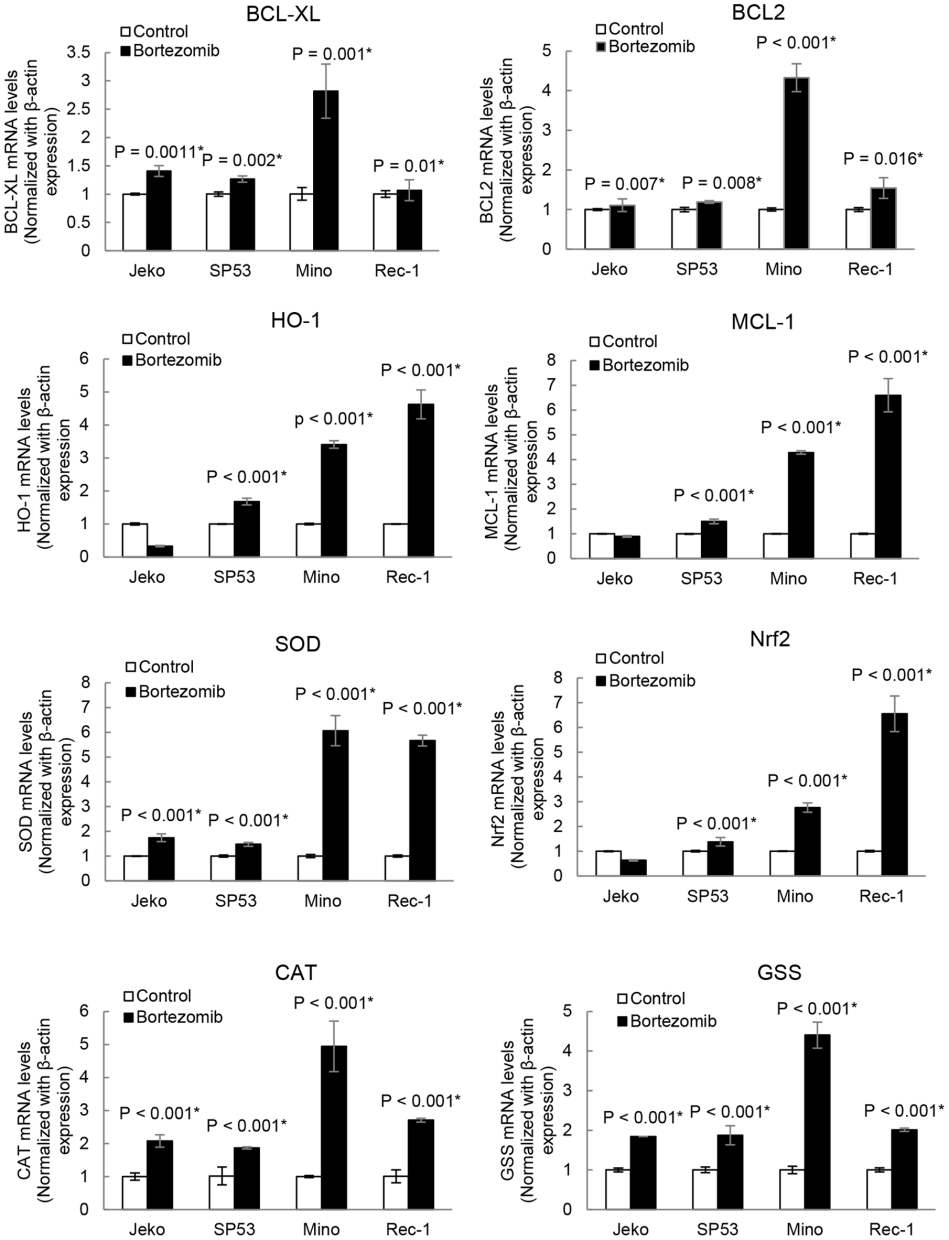
BACH2 nuclear translocation suppresses expression of pro-survival and anti-oxidative genes in bortezomib sensitive cell lines (Jeko and SP53) but not in bortezomib resistant cell lines (Mino and Rec-1). Jeko, SP53, Mino, Rec-1 cells were treated with bortezomib (20 nM) for 24 h and total RNA was isolated by an RNA extraction kit. Purified RNA (25 ng) was used for cDNA synthesis using oligo dT primers. cDNAs of control and treated samples were used to determine the level of gene expression changes after bortezomib treatment. Expression level for each gene (*HO-1, MCL-1, Nrf2, CAT* and *Gss*) was normalized with β-actin expression. Data were expressed as mean ± SD of four experiments done in duplicates or triplicates for each gene and plotted as a relative fold of decrease or increase in bortezomib-treated samples compared to the respective gene expression in untreated controls.

Since BACH2 is a transcriptional repressor and binds to MARE/ARE sequences in DNA [21], [22], we hypothesized that BACH2 could suppress the expression of genes that are involved in anti-oxidative defense upon translocation. These collective features provide bortezomib resistant cells a prosurvival advantage under a stressed condition. BACH2 regulated prosurvival genes are not fully characterized except heme oxygenase-1 (HO-1), which has been shown to protect the cells against oxidative stress and apoptosis [33]. Therefore, we selected additional four anti-oxidative genes [Nuclear factor erythroid 2-related factor 2 (*Nrf2*), GSH synthase (*Gss*), superoxide dismutase (*SOD*), catalase (*CAT*)] and four anti-apoptotic genes [HO-1, MCL-1, BCL2 and Bcl-xL] based on their approved role in redox mitigation and anti-apoptosis [33]–[35]. Real time PCR results revealed that bortezomib-treated Jeko and SP53 cells expressed lower levels of anti-oxidative genes *Nrf2*, *Gss* and CAT and anti-apoptotic genes *HO-1* and *MCL-1* as compared to bortezomib treated Mino and Rec-1 cells (**Figure 6**). In contrast, bortezomib treatment in Mino and Rec-1 induced significant upregulation of *Nrf2, Gss, CAT, HO-1* and *MCL-1.* Thus, these results suggest that bortezomib induced nuclear translocation of BACH2 in Jeko and SP53 cells triggers repression of genes that are involved in anti-apoptosis and anti-oxidative defense and lower the threshold for induction of apoptosis.

### PI3K inhibitors increase retention of BACH2 in the nucleus

**Figure 7:**
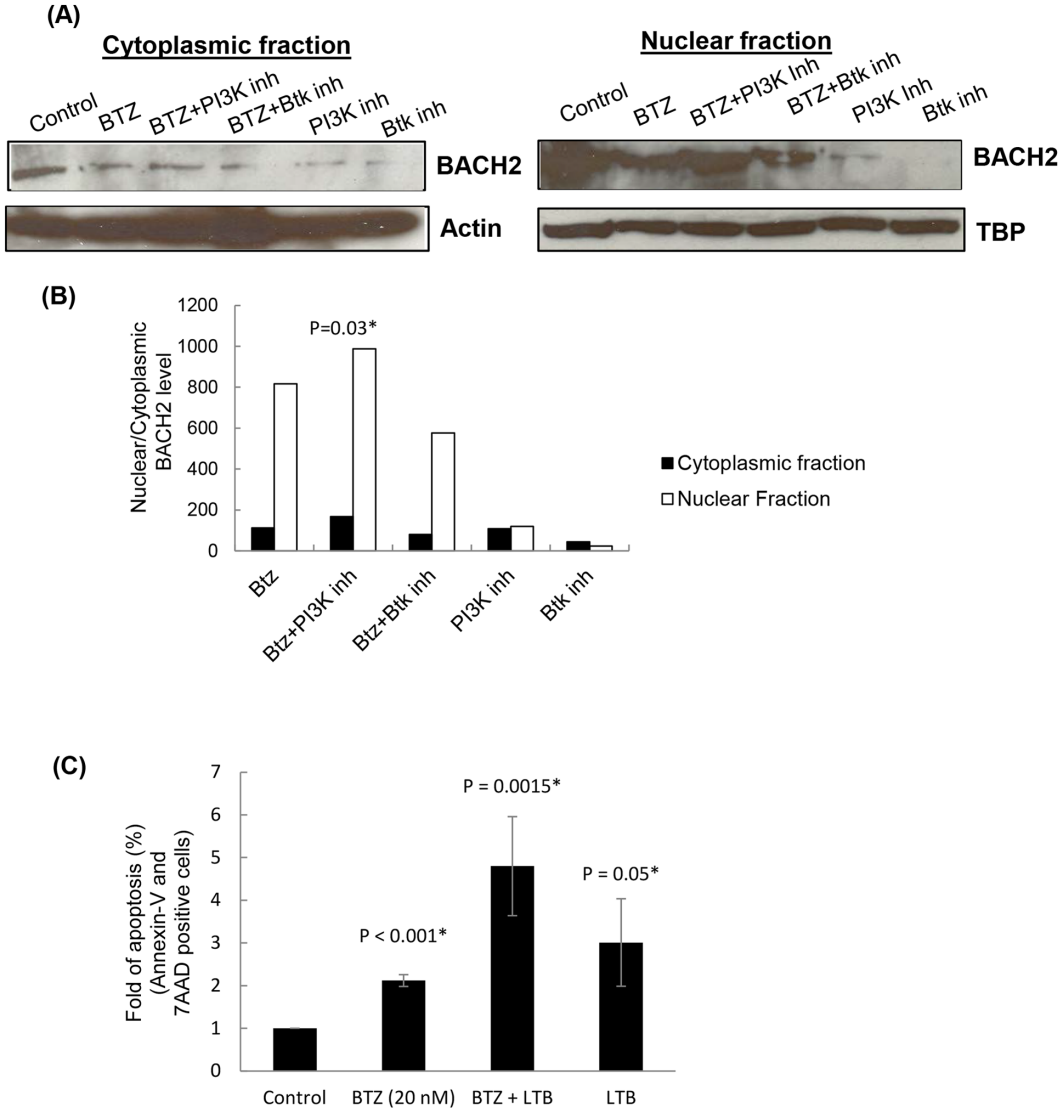
PI3K inhibitors (LY-294002) increase nuclear translocation of BACH2 and enhance MCL cytotoxicity. (**A**) Effect of PI3K inhibitors on nuclear localization of BACH2 was analyzed by immunoblottings in Jeko cells. Cells were pretreated with PI3K inhibitors (LY294002, 50 µM) or Bruton's tyrosine kinase inhibitors (PCI-32765, 1 µM/1 h) followed by bortezomib treatment (20 nM/24h). Bruton's tyrosine kinase inhibitor (PCI-32765) was used as a control, which did not show increased nuclear translocation of BACH2. Cytoplasmic and nuclear fractions for each sample were separated by hypotonic method using a kit. TBP was shown as loading control for nuclear proteins. Controls are Jeko cells without any treatments. 293T cells were used as positive control for BACH2. Data represent four independent experiments. (**B**) BACH2 levels in each sample were plotted in the graph. (**C**) Inhibition of nuclear export of BACH2 by leptomycin-B (LTB) enhanced the bortezomib-induced apoptosis in Jeko cells. Apoptosis was determined by Annexin-V/7-AAD dual staining method using FACS. Error bars indicate the standard error of the means (SEM) from two independent experiments. Results are expressed as an increase in fold of percentage apoptosis compared to control. LTB treatment was given 6 h prior to BTZ treatment for 24 h.

Our results clearly demonstrated that oxidative stress induced by bortezomib in Jeko and SP53 cells caused nuclear translocation of BACH2 which resulted in repression of antiapoptotic and antioxidative genes. Since earlier reports have indicated that PI3K phosphorylates BACH2 and maintains its cytoplasmic localization [35], we tested whether specific kinase inhibitors would inhibit BACH2 translocation to the cytoplasm and sensitize MCL cells for bortezomib-induced cytotoxicity. Immunoblotting of nuclear fraction of Jeko cells treated with either bortezomib alone or in combination with PI3K inhibitor (LY294002) showed that inhibiting PI3K in bortezomib-treated Jeko cells resulted in increased retention of BACH2 in the nucleus compared to bortezomib only treatment (**Figures 7A–B**). Leptomycin-B (LTB), a specific inhibitor of exportin-1 protein inhibits nuclear export of proteins [21]. Using LTB, we tested the possibility that nuclear accumulation of BACH2 can increase the bortezomib-induced cytotoxicity in MCL cells. Due to its maximum level of BACH2 among MCL cells, we selected Jeko cells to test the effect of bortezomib in LTB-pretreated cells. Results showed that pretreatment of LTB significantly enhanced the percentage of bortezomib-induced apoptosis compared to only bortezomib or LTB treatment (**Figure 7C**).

### PI3 kinase inhibitors synergistically increase bortezomib cytotoxicity in MCL

**Figure 8:**
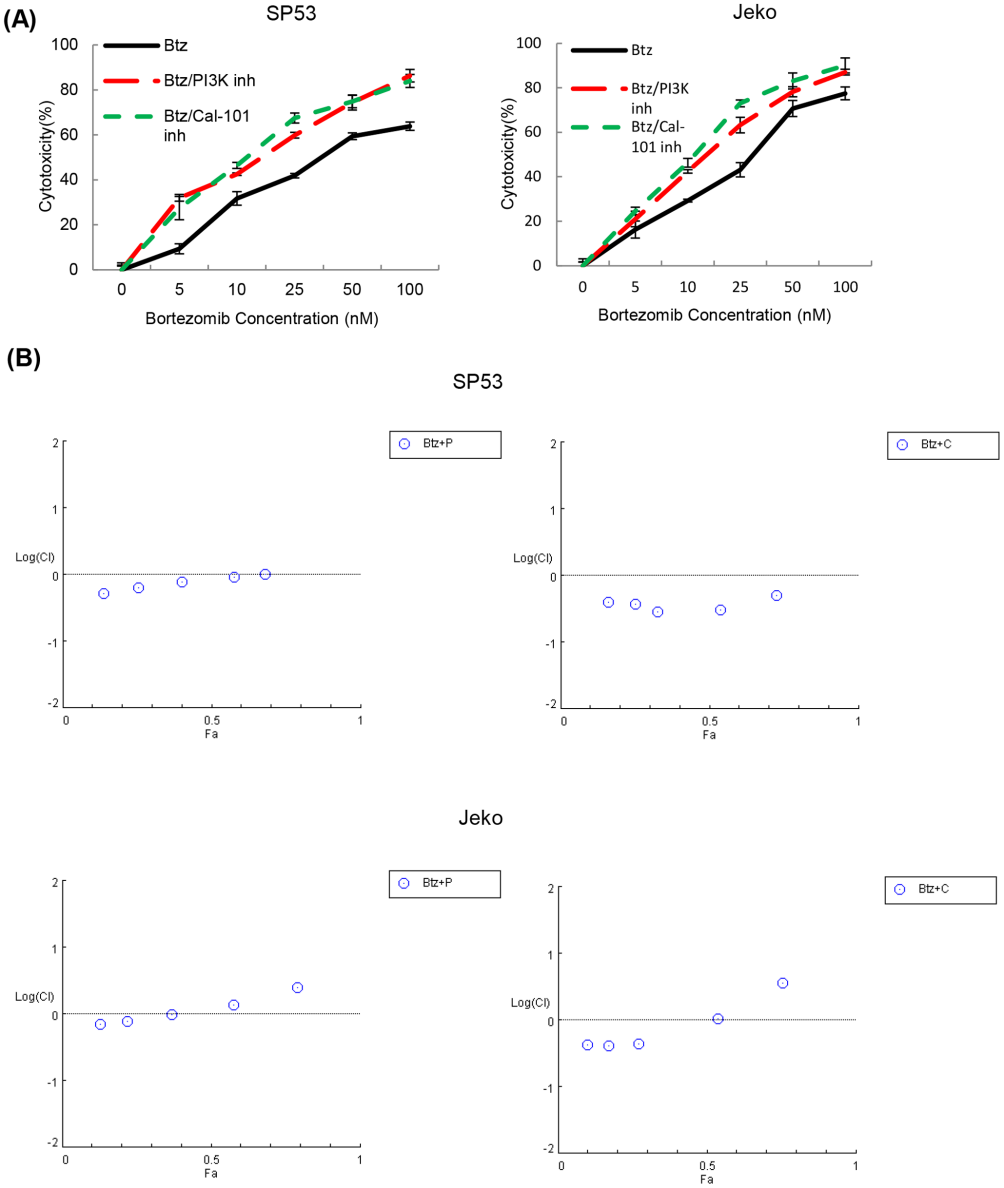
PI3 kinase inhibitors synergistically increase the cytotoxicity of bortezomib in MCL cell lines. (**A**) The addition of PI3 kinase inhibitors with bortezomib significantly increased the cytotoxicity in MCL cells. Cell viability of MCL cells (Jeko and SP53) was measured after overnight incubation with PI3 kinase inhibitors (20 µM) and bortezomib (0–100 nM). Data represent three independent experiments. (**B**) The synergic cytotoxic effects of PI3 kinase inhibitors and bortezomib were determined using the combination index (CI) based on the data from cell viability assays. CI plots were generated using the CompuSyn software (ComboSyn, NJ, USA) according to the Chou-Talalay method. F.a. is Fraction affected.

Cytotoxic relevance of the preceding observation was further studied by measuring viability of Jeko cells treated with bortezomib in combination with PI3 kinase inhibitors (LY-294002 or CAL-101). Cell viability was measured using a fluorometric assay. Results clearly showed that treatment of PI3 kinase inhibitors enhanced bortezomib-induced cytotoxicity in Jeko cells compared to their individual treatments (**Figure 8A** **, Figure S2**). The synergistic cytotoxic effects of bortezomib and PI3 kinase inhibitors were analyzed by combination index (CI) plots, based on the Chou and Talalay method. When Jeko MCL cells were treated with bortezomib and PI3 kinase inhibitors, the log (CI) values were mainly less than 0, suggesting synergistic responses (**Figure 8B**).

## Discussion

The molecular basis for differential clinical responses to bortezomib treatment is poorly understood and may be both disease and patient specific. A rationale for the development of bortezomib has been its ability to inhibit the nuclear factor-kB (NF-kB) pathway through reduced proteasomal degradation of the NF-kB inhibitor IkBα [11]. However, recent studies, including our own, showed NF-kB activities in some MCL patients and MM (multiple myeloma) patients are resistant to bortezomib treatment [25], [36]–[38]. In addition, effectiveness of bortezomib in other tumor models that are not exclusively dependent on NF-kB activation for their malignant progression suggests that NF-kB inhibition may not be the only mechanism important for cytotoxic activity of bortezomib. Furthermore, expression of plasmacytic markers in both MCL cell lines and primary cells of MCL patients have also been shown to be associated with inferior cytotoxic response to bortezomib therapy [25], [39]. Thus, these findings indicated the existence of additional mechanisms responsible for the intrinsic or acquired resistance of MCL cells to bortezomib.

Inhibition of the proteasome severely disrupts protein homeostasis and leads to rapid accumulation of polyubiquitinated proteins in the cytosol. This increase in protein load triggers an oxidative stress response which has been shown to activate programmed cell death by multiple intracellular mechanisms [17]–[19]. In the present study, we focused on determining the role of an oxidative stress sensitive-B-cell specific transcription factor, BACH2, in bortezomib response of MCL cells. To represent the heterogeneous spectrum of MCL disease, we selected four cell lines Jeko, SP53, Mino and Rec-1 for the present study. Significantly lower expression of BACH2 -mRNA as well as -protein in these bortezomib resistant MCL cell lines compared to bortezomib sensitive MCL cells as well as patient cells (**Figure 1**) suggested that BACH2 might function as a tumor suppressor in MCL cells. Our results also showed that bortezomib-treated cells exhibited a higher level of BACH2 compared to control. Therefore, these results allowed us to interpret that MCL cells seemed to possess a different post-translational mechanism to regulate cellular levels of BACH2 than normal B-cells. However, there could be multiple mechanisms such as methylation status, copy number, or promoter activity of BACH2 gene that might account for the decreased expression of BACH2 in MCL cells. It is interesting to mention that bortezomib responsiveness of Jeko, SP53, Mino and Rec-1 showed a correlation to their basal BACH2 protein levels. Following this, and the observed role of oxidative stress involvement in anti-MCL activity of bortezomib, (**Figures 2A–B**), we sought to elucidate the BACH2-based mechanism that might play a central role in differential response of MCL cells to bortezomib-induced cytotoxicity and apoptosis.

**Figure 9:**
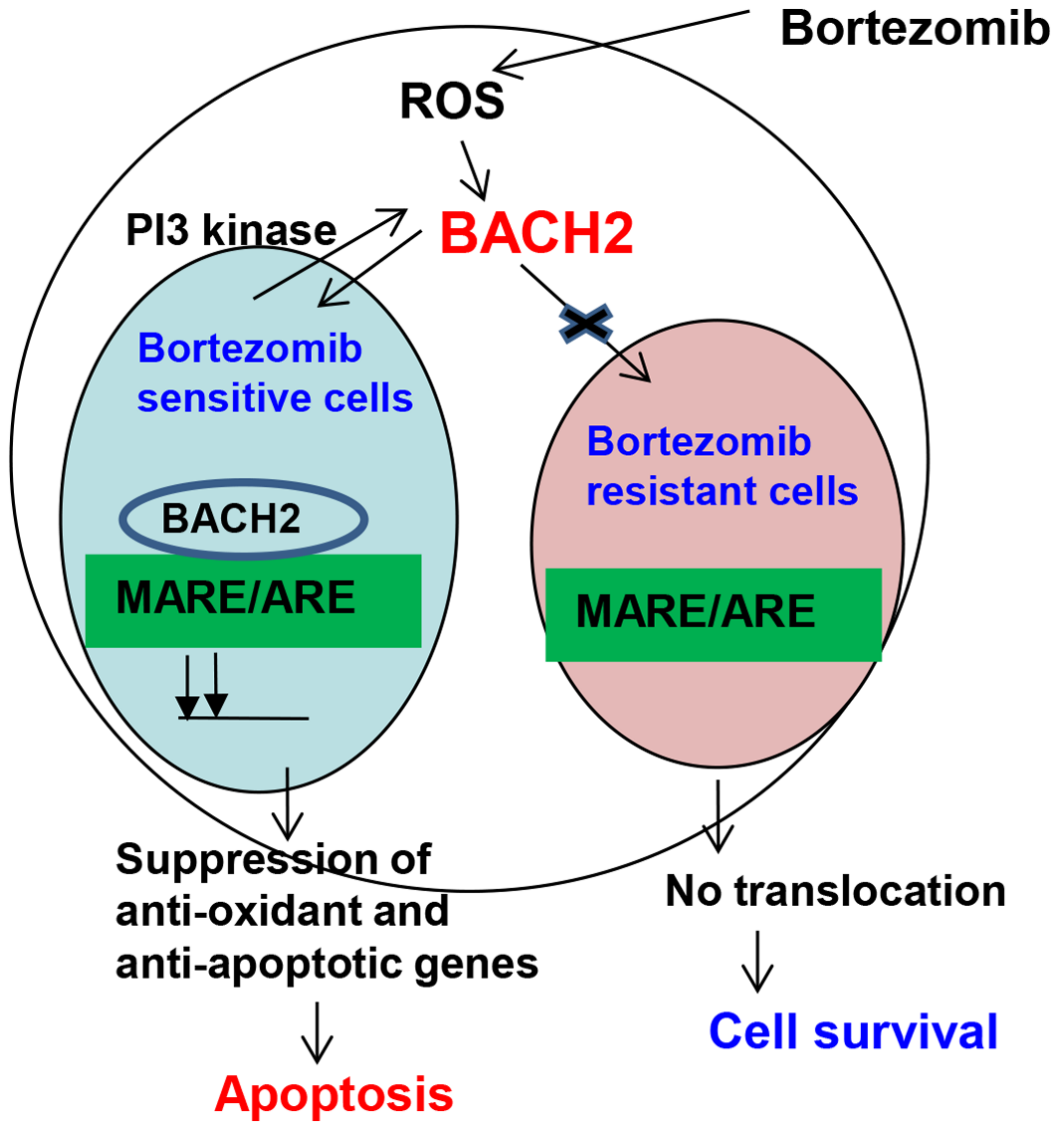
BACH2 subcellular localization determines cell fate in response to oxidative stress. BACH2 subcellular localization determines oxidative stress responses in MCL. Increased levels of BACH2 in the nucleus suppress anti-oxidant and anti-survival genes in the bortezomib sensitive cell lines Jeko and SP53, leading to cell death. However, in the bortezomib resistant cell lines Mino and Rec-1, BACH2 does not translocate into the nucleus. Nuclear export of BACH2 is leptomycin B sensitive and mediated by PI3 kinase phosphorylation.

BACH2 is localized in the cytoplasm through its C-terminal cytoplasmic localization signal (CLS) activity. The CLS directs leptomycin-B sensitive and exportin-1 dependent nuclear export. Reports have shown that oxidative stress aborts CLS activity and induces nuclear accumulation of BACH2 [23], [40]. Employing confocal microscopy and immunoblotting, our results have also showed nuclear translocation of BACH2 in Jeko cells treated with bortezomib (**Figures 3B** **and** **4A**). Kamio et al. reported diffused BACH2 fluorescence in the nucleus of transfected Raji-cells upon treatment with ROS-genic cytotoxic agents [23]. Thus these studies suggest that BACH2-mediated gene regulatory sub-nuclear structure could be cell type-dependent and might involve a complex mechanism with multiple factors. In accordance with the preceding interpretation, a study reported the recruitment of BACH2 upon oxidative stress in promyelocytic leukemia nuclear (PML) nuclear bodies [40]. PML bodies have been shown to be one of the most prominent nuclear substructures implicated in transcription regulation and stress response [41]. Confocal image analysis revealed that bortezomib treatment resulted in a higher level of BACH2 in the nucleus of Jeko and SP53. Bortezomib-resistant cells Mino and Rec-1 did not exhibit significant nuclear accumulation of BACH2 (**Figures 5A–B**). These results suggest the existence of a complex intracellular mechanism that seems to control nuclear translocation of BACH2. Based on these results, nuclear translocation of BACH2 seemed to be an important mechanism to determine the bortezomib response in MCL cells (**Figure 9**).

BACH2 binds to the Maf recognition element (MARE) as a heterodimer with small Maf proteins and usually inhibits target genes. The MARE is most closely related to antioxidant response element (ARE) after AP-1–binding DNA sequences. ARE regulates the expression of several genes involved in oxidative stress defense [23]. This redox defense is mediated by thiol-rich molecules such as glutathione synthesized by glutathione synthase (GSS), H_2_O_2_ neutralizing enzymes such as catalase (CAT), and heme oxygenase 1 (HO-1), which protects cells from oxidative stress by generating a potent radical scavenger bilirubin. Redox stimuli have been shown to induce nuclear localization of BACH2 where it acts as a transcriptional repressor of ARE/MARE sequences [23]. Based on the following information, we decided to study changes in expression of antioxidative genes (*Gss, CAT, Nrf2*) in combination with certain well characterized antiapoptotic genes (*HO-1* and *MCL-1*). RT-PCR results demonstrated that bortezomib treatment significantly upregulated the expression of Nrf2, Gss, CAT, HO-1 and MCL-1 genes in Mino and Rec-1. In contrast, Jeko and SP53 exhibited either decreased or insignificant changes in the expression of these antioxidative and antiapoptotic genes after bortezomib treatment. In combination with the results of bortezomib-induced nuclear translocation of BACH2 in Jeko and SP53, gene expression data suggested that bortezomib-induced oxidative stress activated BACH2 nuclear translocation thus repressed the expression of antioxidative and antiapoptotic genes, which seems to culminate in lowering the threshold for the induction of apoptosis. However, in Mino and Rec-1, confocal study showed insignificant translocation of BACH2 that might allow the recruitment of transcriptional activators of ARE/MARE sequences, resulting in the up regulation of Nrf2, Gss, CAT and HO-1 genes. This suggests a role for Bach2 as an intracellular molecular switch that processes and transduces an oxidative stress signal from cytoplasm to the nucleus to eliminate the aged or defective cells from circulation. We discovered that inhibition of PI3K induced significant nuclear accumulation in bortezomib treated Jeko cells (**Figures 5A and B**), indicating synergistic cytotoxicity. The role of PI3K in the present study indicates additional mechanisms employed by MCL cells to develop resistance against oxidative stress as compared to CML cells. Collectively, these findings indicate that the combination treatment of PI3K inhibitors with bortezomib could have synergistic cytotoxic effects on MCL.

## Supporting Information

Figure S1 BACH2 levels in different MCL cell lines and patient cells. (**A**) Protein levels of BACH2 in whole cell lysates of indicated MCL were determined by immunoblotting. Actin was used as a protein loading control. Raji cell lysate was used as a negative control. (**B**) IC50 values between bortezomib resistant samples and bortezomib sensitive samples. (**C**) BACH2 mRNA levels were measured by real-time PCR using different MCL patient samples.
(PDF)

Figure S2 PI3 kinase inhibitors synergistically increase bortezomib cytotoxicity in MCL. PI3 kinase inhibitors synergistically enhanced bortezomib induced cytotoxicity in Jeko and SP53 MCL cells.
(PDF)
