# Supplementary figures and images for: Nuclear Translocation of B-Cell-Specific Transcription Factor, BACH2, Modulates ROS Mediated Cytotoxic Responses in Mantle Cell Lymphoma
Source: PLoS One. 2013 Aug 2;8(8):e69126. doi: 10.1371/journal.pone.0069126 (PMC3732253; doi:10.1371/journal.pone.0069126)

Supplemental Figure 1.

Chen et al.

(A)

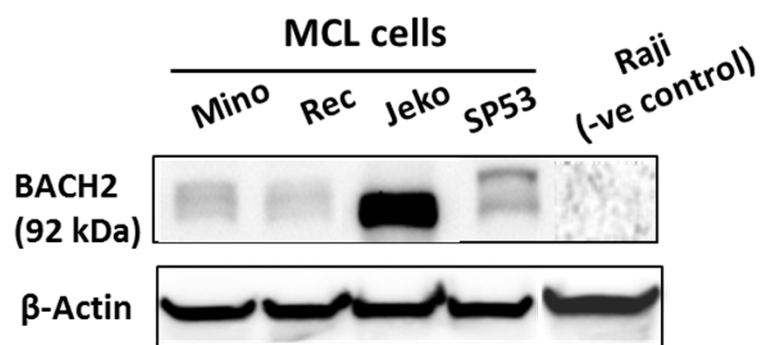

(B)

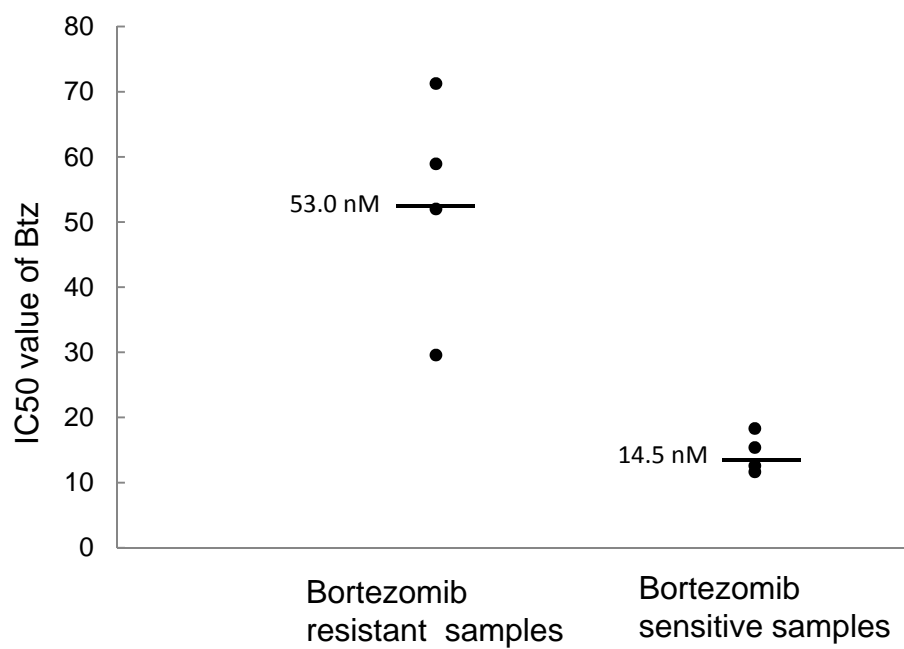

(C)

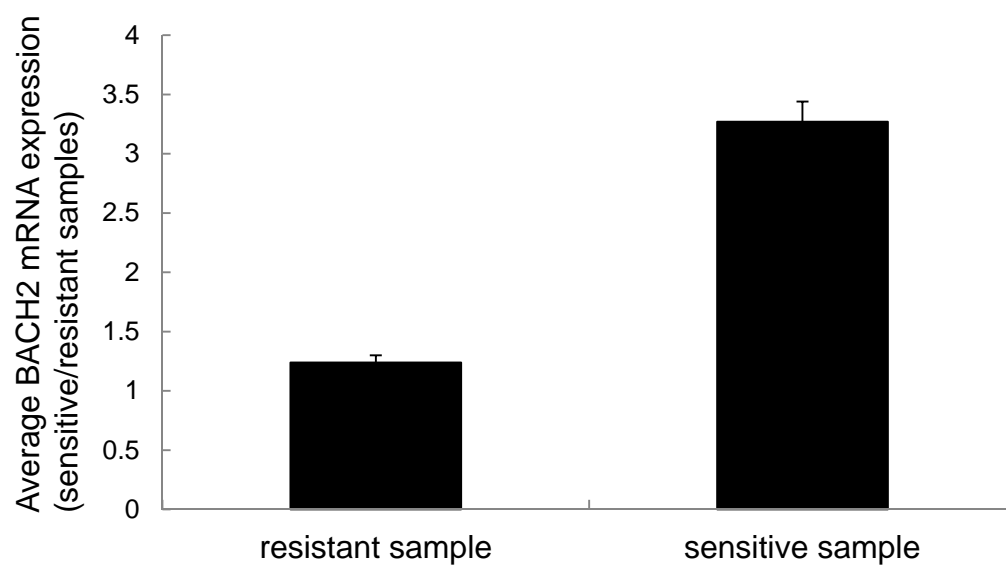

Supplement: Figure S1 — BACH2 levels in different MCL cell lines and patient cells. (A) Protein levels of BACH2 in whole cell lysates of indicated MCL were determined by immunoblotting. Actin was used as a protein loading control. Raji cell lysate was used as a negative control. (B) IC50 values between bortezomib resistant samples and bortezomib sensitive samples. (C) BACH2 mRNA levels were measured by real-time PCR using different MCL patient samples. (PDF) [file pone.0069126.s001.pdf]

Supplemental Figure 2.

**SP53**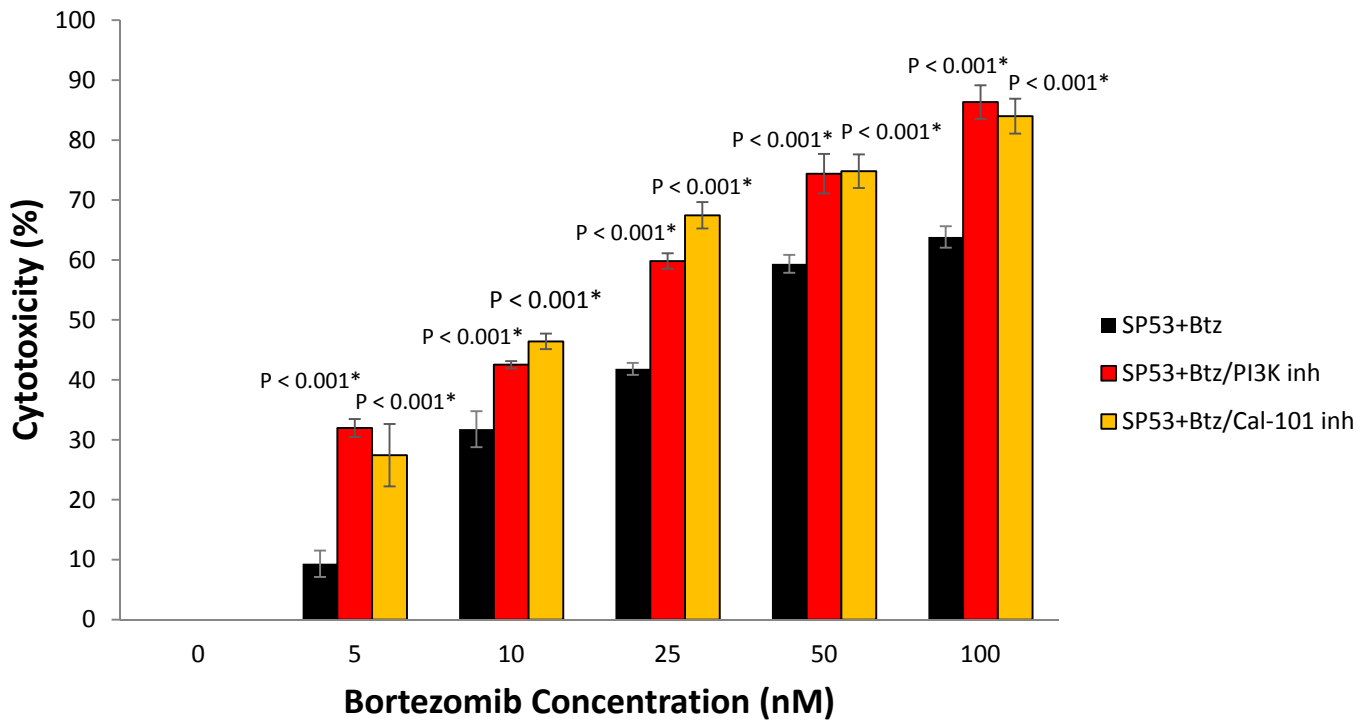**Jeko**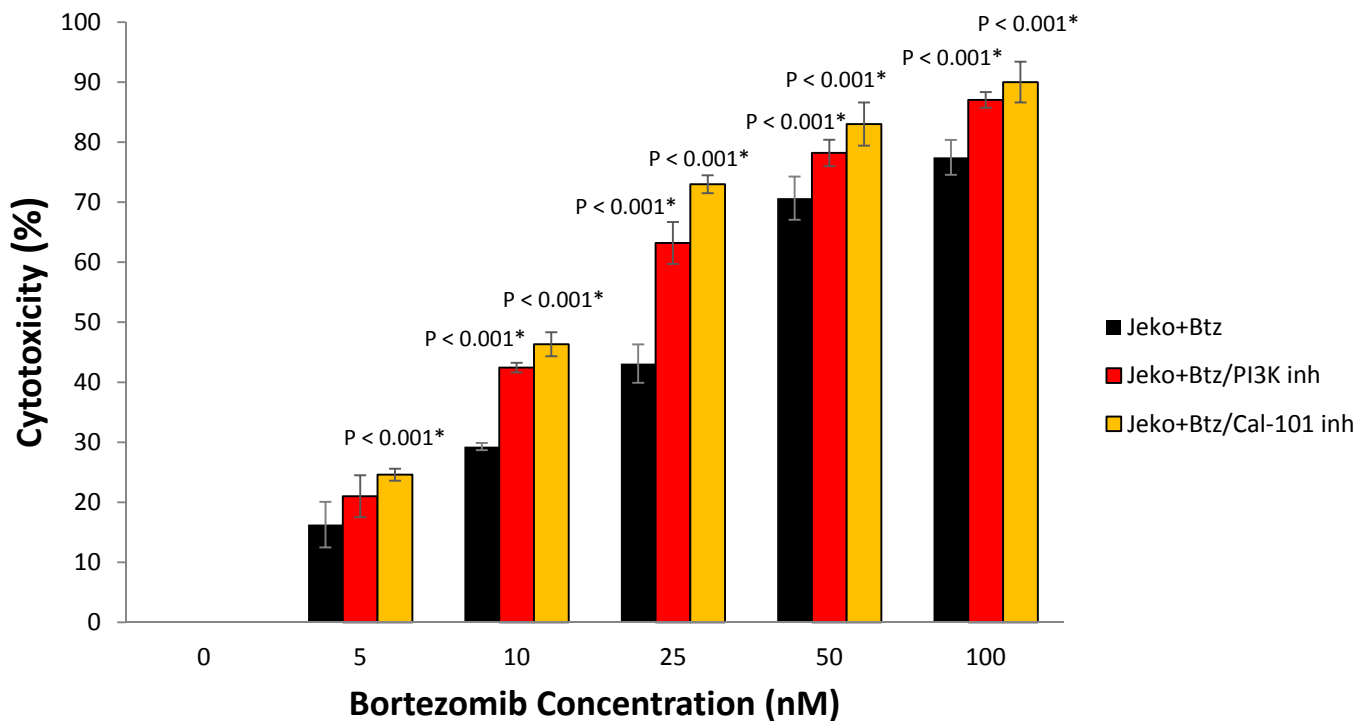

Supplement: Figure S2 — PI3 kinase inhibitors synergistically increase bortezomib cytotoxicity in MCL. PI3 kinase inhibitors synergistically enhanced bortezomib induced cytotoxicity in Jeko and SP53 MCL cells. (PDF) [file pone.0069126.s002.pdf]
